# Big Five Personality Traits are Associated with Tinnitus Improvement Over Time

**DOI:** 10.1038/s41598-019-53845-4

**Published:** 2019-12-03

**Authors:** Jorge Simões, Winfried Schlee, Martin Schecklmann, Berthold Langguth, Daria Farahmand, Patrick Neff

**Affiliations:** 1 Department of Psychiatry and Psychotherapy, University of Regensburg, Regensburg, Germany; 2 University Research Priority Program ‘Dynamics of Healthy Aging’, University of Zurich, Zurich, Switzerland

**Keywords:** Risk factors, Disability

## Abstract

Previous studies have shown that personality traits are related to tinnitus distress as measured by the Tinnitus Handicap Inventory (THI) and the Tinnitus Questionnaire (TQ). However, little is known about the role of personality on tinnitus distress over time. We collected the THI and the TQ of 388 patients who visited a tertiary tinnitus clinic between 2012 and 2017, and who filled in a survey with the same questionnaires plus the Big Five Index 2 in 2018. We used personality traits and facets to predict tinnitus distress cross-sectionally and longitudinally. Neuroticism, extraversion, agreeableness, age and gender were significant predictors of the THI and TQ scores in cross-sectional linear regression setups. Next, based on previous literature, we clustered patients in three groups based in the difference THI and TQ between the two assessments: “clinically improved”, “clinically stable” and “clinically worsened”. The patients in the “clinically improved” and “clinically stable” groups scored statistically significantly lower in neuroticism and higher in extraversion than patients in the group “clinically worsened”. Our results suggest that personality is associated with tinnitus distress over time and could be used to statistically distinguish patient groups with clinically relevant changes of tinnitus distress.

## Introduction

Tinnitus is a condition characterized by the subjective perception of sounds without an external source^1^ that affects between 10 and 15% of the population in western societies^2^. Currently, there is no treatment available to reliably and effectively suppress the phantom perception in chronic (i.e., lasting more than six months)^2^, idiopathic presentation of tinnitus. In those cases, treatment focus on the condition’s management, but response to treatments vary considerably across patients. It is yet not fully understood why clinically available treatments do not reliably and effectively suppress the distress associated with tinnitus, but heterogeneity may be a relevant explaining factor^3^. Previous studies suggested that treatments’ low evidence levels could be explained by individual factors^3–5^, and whether personality could be one of such factors is not yet known.

The construct of personality can be described as the individual profile in characteristic patterns of thinking, feeling and behaving^6^. Personality research has implications in a wide range of topics, including ones related to tinnitus such as memory^7^ and sleep^8^. Several models have been developed to characterize and quantify different personality aspects, such as the five factor model (FFM, also known as the “Five Factor Model” or “Big Five Personality Traits”). The model quantifies five traits of personality, namely agreeableness, conscientiousness, extraversion, neuroticism, and openness. John and Soto^9^ recently developed the Big Five Index 2 (BFI-2), a revised version of the original questionnaire that quantifies each of those traits. This reviewed version includes features such as robust hierarchical structure, control for acquiescent responding, fidelity, and increased predictive power, while retaining key features of the original BFI such as its ease of understanding and conceptual focus^9^. The BFI-2 also comprises 15 facets, or subtraits, describing different aspects of each trait^10^. A brief description of the five traits and 15 facets can be found in Table 1, and a more in-depth analysis of the BFI2 and its constructs is available^9^. Apart from the childhood and teenage years, personality is believed to be stable over time, especially after the age of 30^11^. It is also known that the stability of traits decreases with longer retest periods. However, previous studies showed high stability of personality traits over time, with scales presenting r = 0.77 and r = 0.73 in the 6 and 12 years retest interval period^12^.

**Table 1:** Example items of facets in the BFI-2.

Traits and Facets	Example items
**Neuroticism**	
Anxiety	(19) Can be tense; Rarely feels anxious or afraid (R)
Depression	(9) Stays optimistic after experiencing a setback (R); (39) Often feels sad
Emotional Volatily	(44) Keeps their emotions under control (R); Is temperamental, gets emotional easily
**Extraversion**	
Sociability	(1) Is outgoing, sociable; (46) Is talkative
Assertiveness	(6) Has an assertive personality; (51) Prefers to have others take charge (R)
Energy level	(11) Rarely feels excited or eager (R); (41) Is full of energy
**Agreeableness**	
Compassion	(2) Is compassionate, has a soft heart; (17) Feels little sympathy for others (R)
Trust	(37) Is sometimes rude to others (R); (52) Is polite, courteous to others
Respectfulness	(12) Tends to find fault with others (R); (27) Has a forgiving nature
**Openness**	
Aesthetic Sensitivity	(35) Values art and beauty; (50) Thinks poetry and plays are boring (R)
Intelectual Curiosity	(25) Avoids intellectual, philosophical discussions (R); (40) Is complex, a deep thinker
Creative imagination	(15) is inventive, finds clever ways to do things; (30) Has little creativity (R)
**Conscientiousness**	
Organization	(3) Tends to be disorganized (R); (33) Keeps things neat and tidy
Productiveness	(8) Tends to be lazy (R); (38) Is efficient, gets things done
Responsibility	(13) Is dependable, steady; (28)

(R) indicates reversed-keyed items.

Looking at tinnitus specifically, Langguth and colleagues^13^ used the FFM to describe the personality traits of tinnitus patients. The authors found that tinnitus patients tend to score higher in neuroticism, and lower in agreeableness. Additionally, a recent scoping review^14^ suggested that personality traits, such as high neuroticism and low extraversion, are common hallmarks of tinnitus patients. Previous works have investigated the prevalence of of the “type D” personality and tinnitus. The type D personality is characterized by both social inhibition and neuroticism, and has been shown to be prevalent in tinnitus patients^15,16^. More recently, Strumila and colleagues^17^ investigated the association between personality, environment, anxiety, depression and distress. Overall, the authors found that anxiety and depressive states were associated with all personality traits apart from agreeableness, and that neuroticism was the only statistically significant predictor of tinnitus distress.

However, studies assessing the putative role of tinnitus distress longitudinally are scarce. Recently, Kleinstäuber and colleagues^18^ investigated the role of personality traits on internet-delivered cognitive behavior therapy (iCBT) in chronic tinnitus patients. The results indicated that different traits can predict the outcome of an iCBT intervention after different time periods (e.g., 3, 6, 12 months after treatment), underscoring the often overlooked influence of personality on treatment outcomes in tinnitus. However, some questions remain unanswered: It is yet not clear if the effects of personality can predict tinnitus-related distress over time, disregard of whether a patient tried any type of treatment or not. It is also unclear whether personality mediates the outcome of psychological-based interventions or, in general, mediates all kinds of tinnitus-related interventions. From a clinical perspective, these open questions are of utmost importance to better understand differences in clinically relevant changes of tinnitus symptomatology. In the study at hand, we aimed at (1) replicating the previous results obtained by Langguth and colleagues^13^, but with a larger sample size; (2) investigating which facets of relevant personality traits account for tinnitus distress; (3) investigating the role of personality traits on tinnitus distress over time; and, of central interest, (4) evaluating whether such traits may be of clinically relevance to the treatment response.

Our hypotheses were: (1) neuroticism correlates positively with tinnitus distress over time (i.e., the higher neuroticism is, the lower distress tends to decline over time), whereas (2) extraversion correlates negatively with tinnitus distress over time; (3) neuroticism correlates positively with changes in distress over time, and (4) neuroticism and extraversion inform differences in clinically relevant grading of the tinnitus distress questionnaires.

## Results

### Cross-sectional analysis of personality traits and facets

**Figure 1:**
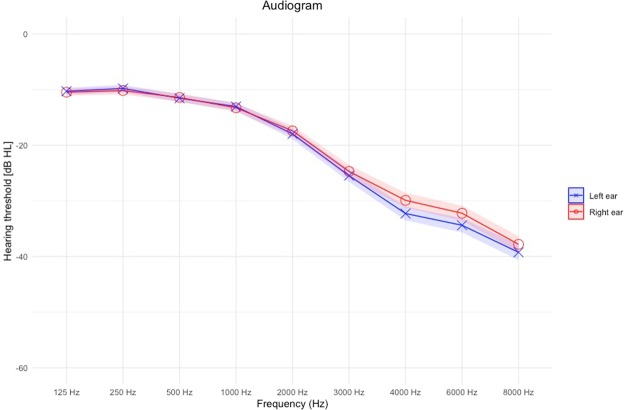
Audiogram of our sample. Ribbons represent the standard error.

Demographics and tinnitus characteristics of our sample can be found in Table 2, and the audiogram of our sample is presented in Fig. 1. First, we applied multiple linear regressions with personality traits, age, and gender as independent variables and the scores of THI and TQ at T2 as dependent variables. Table 3 shows both statistical models. Both models showed a statistically significant negative association between extraversion and the questionnaire outcomes, while gender, agreeableness and neuroticism showed a positive association with the questionnaires outcomes. As gender was encoded as a categorical variable, dummy variables were created for each of its levels. Here, the gender “female” was used as reference. Therefore, our model estimated 6 and 4 points (ie., the coefficients obtained from the models) increase in the THI and TQ respectively among men after controlling for the personality traits and age. Age was a statistically significant predictor of the TQ but not of the THI. Moreover, the models could explain 39% of the variance (R2 = 0.39, F(7,334) = 31.98, p < 0.001) with the THI as the dependent variable and 34% of the variance (R2 = 0.34, F(7,336) = 26.02, p < 0.001) with the TQ as the dependent variable.

**Table 2:** Demographics of our sample.

	Frequencies and Means [SD]
Gender (f/m)	146/242
Hearing Loss*	245/152/16
Type of Tinnitus Sound**	232/31/72/46
Laterality of Tinnitus***	50/62/88/57/85/41
Age (years)	55.9 [11.9]
Duration of Tinnitus (months)	154.3 [104.1]
Loudness (1–100)	68.2 [53.3]
THI	49.5 [23.3]
TQ	42.2 [17.7]
Agreeableness	44.5 [5.9]
Conscientiousness	46.3 [7.3]
Extraversion	37.9 [7.8]
Neuroticism	35.7 [8.3]
Openness	39.8 [7.9]

*From left to right: Yes, No, No answer. **From left to right: Tonal, Noise, Crickets, Other. ***From left to right: right ear, left ear, both ears (worse in right), both ears (worse in left), both ears (equally bad), inside the head.

**Table 3:** Linear regression models with personality traits as independent variables and THI and TQ at T2 as dependent variables.

	THI				TQ			
	Coeficients	Std. Error	t value	p value	Coeficients	Std. Error	t value	p value
Intercept	−16.92	14.93	−1.13	0.25	−10.23	12.34	−0.83	0.41
Agreeableness	0.62	0.21	3.06	<**0**.**01**	0.35	0.17	2.06	**0**.**04**
Conscientiousness	−0.25	0.16	−1.54	0.12	−0.14	0.13	−1.05	0.29
Extraversion	−0.47	0.16	−3.02	<**0**.**01**	−0.29	0.13	−2.20	**0**.**03**
Neuroticism	1.56	0.14	10.86	<**0**.**0001**	1.1	0.12	9.16	<**0**.**0001**
Openness	−0.11	0.15	−0.757	0.44	−0.19	0.12	−1.58	0.16
Age	0.07	0.08	0.865	0.39	0.26	0.07	3.75	<**0**.**001**
Gender: male	6.2	2.24	2.76	**0**.**01**	4.08	1.84	2.22	**0**.**03**

Next, we modeled the facets of agreeableness, extraversion and neuroticism as independent variables and the scores from T2 as the dependent variable as these two traits were statistically significant in the previous models. Results are presented in Tables 4, 5 and 6. Regarding the facets of neuroticism, both the models showed a statistical significant association between depression (positive) and emotional volatility and anxiety (negative) with the dependent variable. The effect size of both models was 28% (THI: R2= 0.279, F(5, 336) = 27.4, p < 0.001; TQ: R2= 0.282, F(5, 338) = 27.94, p < 0.001). We did not observe any of the facets of extraversion to be statistically significant in either of the regression setups (THI: R2 = 0.04, F(5, 336), p = 0.002; TQ: R2 = 0.04, F(5, 338) = 0.002). Regarding the facets of agreeableness, “respectfullness” and “trust” were positively and negatively associated with the THI (R2 = 0.05, F(5, 336) = 4.33, p < 0.001), but no facet reached significance in the model with the TQ as dependent variable (R2 = 0.07, F(5, 338) = 6.43, p < 0.0001).

**Table 4:** Linear regression models with the facets of the personality trait “agreeableness” as independent variable and THI and TQ at T2.

	THI				TQ			
	Coeficients	Std. Error	t value	p value	Coeficients	Std. Error	t value	p value
Intercept	34.22	8.72	3.92	**<0**.**001**	14.7	6.85	2.15	**0**.**03**
Compassion	−0.21	1.01	−0.211	0.83	0.05	0.79	0.62	0.95
Respectfulness	−3.28	0.87	−3.78	**<0**.**001**	0.6	0.61	0.98	0.32
Trust	3.45	1.03	3.36	**<0**.**001**	−1	0.77	−1.29	0.19
Age	0.04	0.1	0.44	0.66	0.25	0.08	3.06	**<0**.**01**
Gender: male	6.25	2.68	2.33	0.02	2.62	2.16	7.22	0.22

**Table 5:** Linear regression models with the facets of the personality trait “neuroticism” as independent variable and THI and TQ at T2.

	THI				TQ			
	Coefficients	Std. Error	t value	p value	Coefficients	Std. Error	t value	p value
Intercept	30.87	9.17	3.34	<0.001	12.35	7.3	1.69	0.09
Anxiety	−2.54	0.81	−3.12	**<0**.**01**	−1.6	0.65	−2.743	**0**.**01**
Depression	6.3	0.6	10.43	**<0**.**0001**	4.6	0.48	9.6	<**0**.**0001**
Emotional Volatility	−2.9	0.59	−4.89	**<0**.**0001**	−2.02	0.47	−4.273	**<0**.**0001**
Age	0.01	0.09	0.17	0.87	0.22	0.07	3.05	<**0**.**01**
Gender: male	3.15	2.27	1.386	0.167	2.53	1.8	1.4	0.16

**Table 6:** Linear regression models with the facets of the personality trait “extraversion” as independent variable and THI and TQ at T2.

	THI				TQ			
	Coeficients	Std. Error	t value	p value	Coeficients	Std. Error	t value	p value
Intercept	51.65	80.5	6.42	**<0**.**0001**	28.51	6.41	4.45	**<0**.**0001**
Sociability	−0.16	0.92	−0.18	0.86	−0.27	0.7	−0.37	0.71
Assertiveness	1.11	0.78	1.43	0.15	0.6	0.61	0.98	0.32
Energy Level	−2.27	0.97	−2.34	**0**.**02**	−1	0.77	−1.29	0.19
Age	0.06	0.1	0.57	0.57	0.25	0.08	3.06	**<0**.**01**
Gender: male	3.3	2.7	1.22	0.22	2.62	2.16	7.22	0.22

### Difference in tinnitus distress Between T1 and T2

Next, we modelled a multiple linear regression with personality traits as independent variables and the difference in the THI and TQ between T2 and T1 as dependent variables. Since T1 represents a visit between 2012 and 2017, we added year of visit as an independent variable in the models. This way, we could account for a potential cumulative effect of time over tinnitus distress over time. The results are presented in Table 7. Conscientiousness was the sole personality trait to reach statistical significance (THI: t = −1.98, p = 0.48), whereas the year of the visit was a statistically significant predictor in both models (THI: t = 2.32, p = 0.02; TQ: t = 2.45, p = 0.01). Additionally, we can report statistical trends for neuroticism (t = 1.8, p = 0.07), and for conscientiousness (t = −1.95, p = 0.05) in the TQ. The model with the THI as dependent variable had 5% of the variance explained by the predictors (R2 = 0.046, F(8,325) = 3.05, p < 0.01), whereas the model with the TQ as the dependent variable had 5% of the variance explained (R2 = 0.048, F(6,331) = 3.14, p = 0.001).

**Table 7:** Linear regression models with the personality traits as independent variable and difference between the scores of THI and TQ in T2 − T1.

	THI				TQ			
	Coeficients	Std. Error	t value	p value	Coeficients	Std. Error	t value	p value
Intercept	−11.36	13.63	−0.83	0.4	−21.03	9.76	−2.15	**0**.**03**
Agreeableness	0.12	0.18	0.68	0.49	0.11	0.13	0.89	0.37
Conscientiousness	−0.28	0.14	−1.98	**0**.**048**	−0.2	0.10	−1.95	0.05
Extraversion	−0.17	0.14	−1.21	0.22	0.01	0.1	0.03	0.98
Neuroticism	0.19	0.13	1.43	0.15	0.17	0.09	1.8	0.07
Openess	−0.04	0.13	−0.27	0.79	−0.01	0.09	−0.01	0.99
Year of Visit	1.19	0.52	2.32	**0**.**02**	0.9	0.36	2.45	**0**.**01**
Age	0.11	0.07	1.52	0.13	0.15	0.05	2.75	**<0**.**01**
Gender: male	1.57	2.02	0.77	0.43	2.03	1.44	1.41	0.16

### Personality and clinical significant differences between T1 and T2, and impact on treatment outcomes between T1 and T2

**Figure 2:**
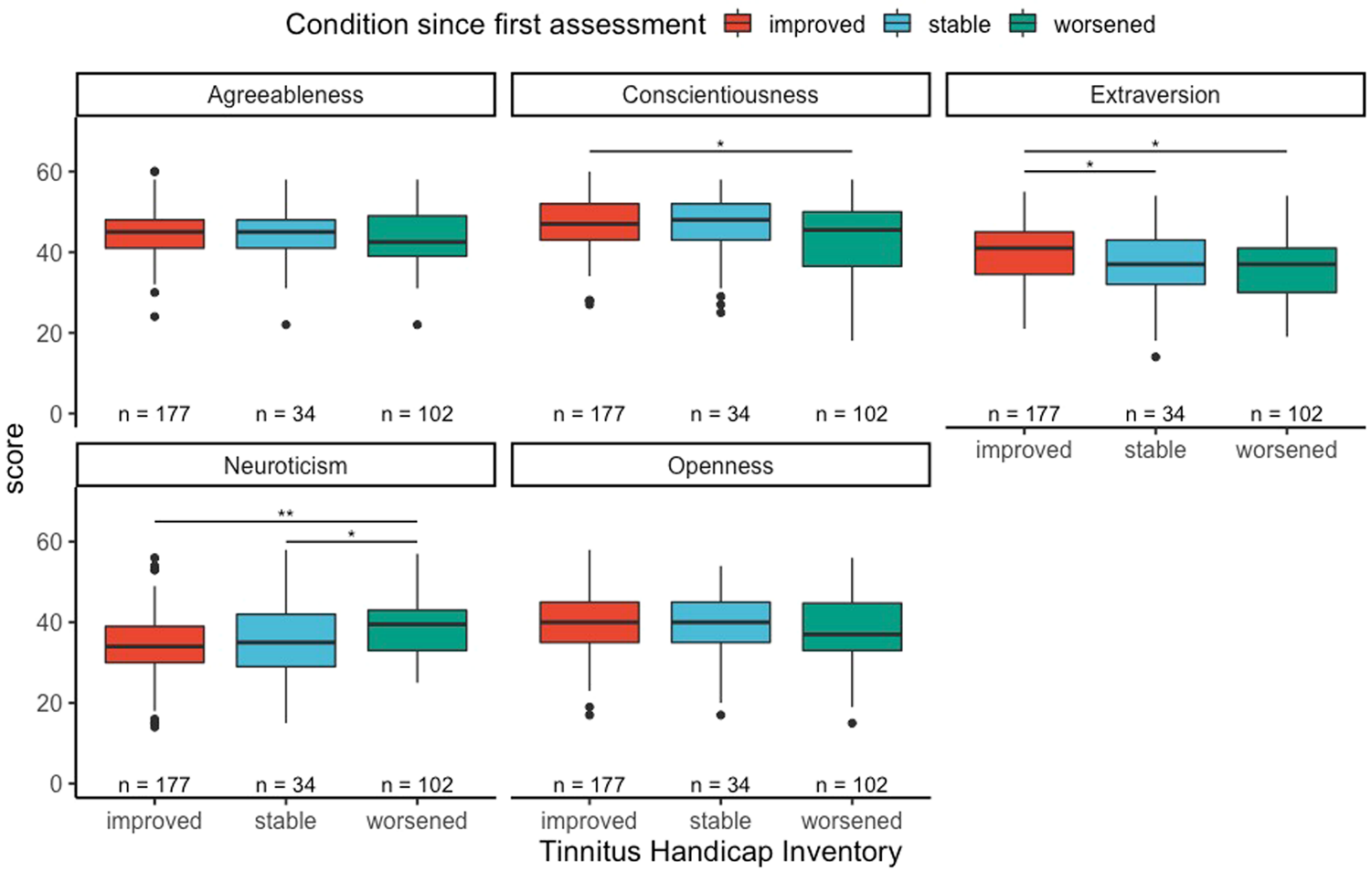
THI change grades and personality traits.

**Figure 3:**
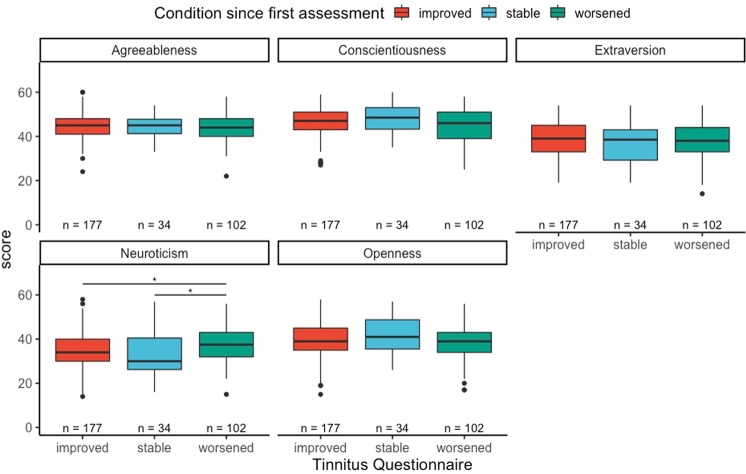
TQ change grades and personality traits.

To further explore the potential role of personality traits on tinnitus distress over time, we grouped patients into three groups based on the difference between the scores of the THI and TQ on T2 − T1. The results are presented in Figs. 2 and 3. Neuroticism was statistically significantly lower in patients in the groups “improved” and “stable” compared to the “worsened” group in the THI (t = −3.3, p-value < 0.01, d = 0.5; t = −2.06, p-value = 0.04, d = 0.34). Likewise, patients in the “stable” groups in the TQ showed statistically significant lower neuroticism scores than the patients binned in the “worsened” group (t = −2.26, p-value = 0.03, d = 0.45). Whereas higher neuroticism was associated with worsening in tinnitus, higher extraversion was associated with improvement in tinnitus distress. Regarding the THI, the patients binned in the “improvement” group scored higher than patients binned in the “stable group” (t = 2.55, p-value = 0.01, d = 0.31) and higher than patients binned in the “worsened” group (t = 2.2, p-value = 0.03, d = 0.34).

**Figure 4:**
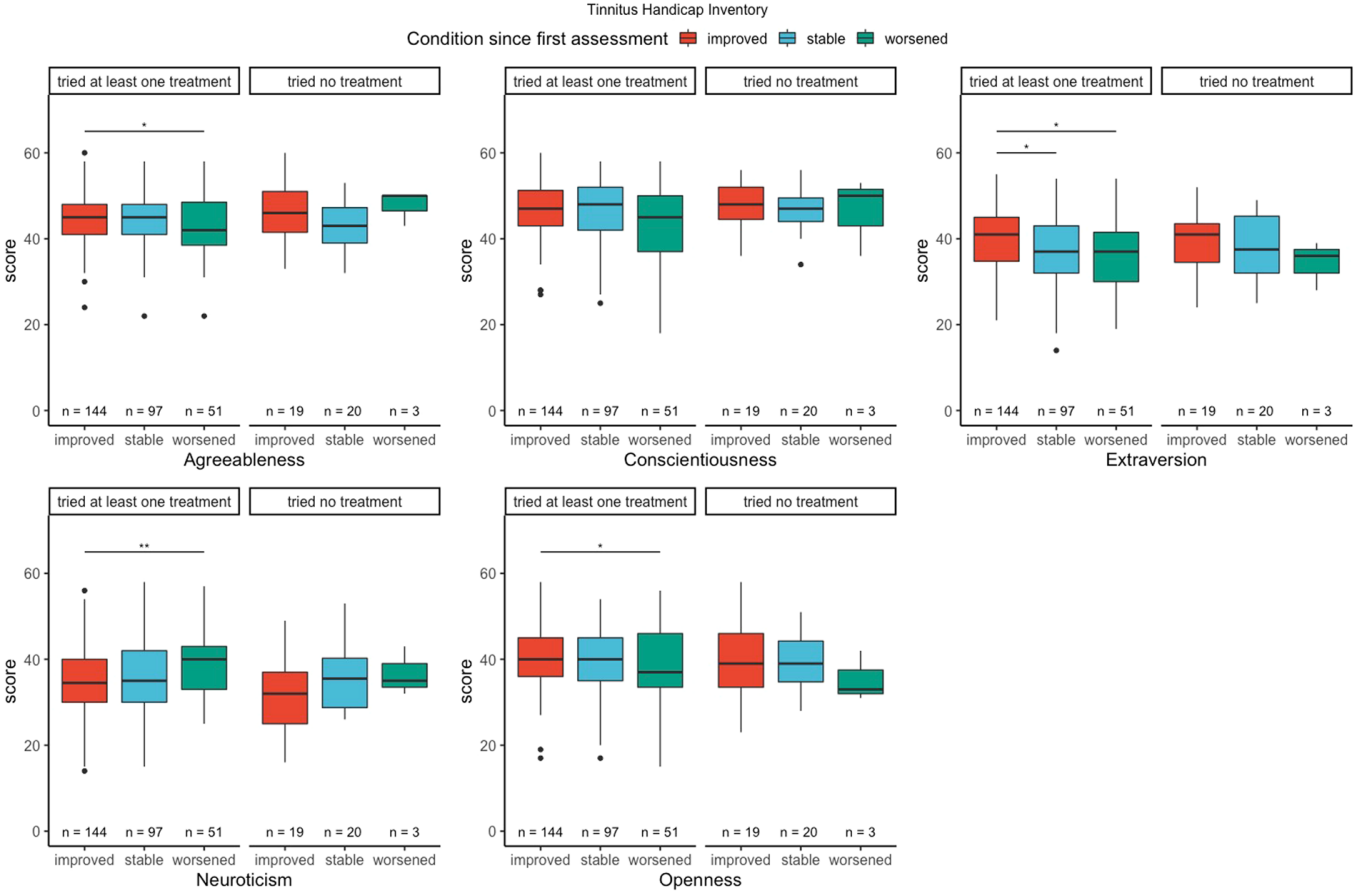
THI change grades and personality traits. Results are reported for patients who tried at least one treatment and patients who tried no treatment.

**Figure 5:**
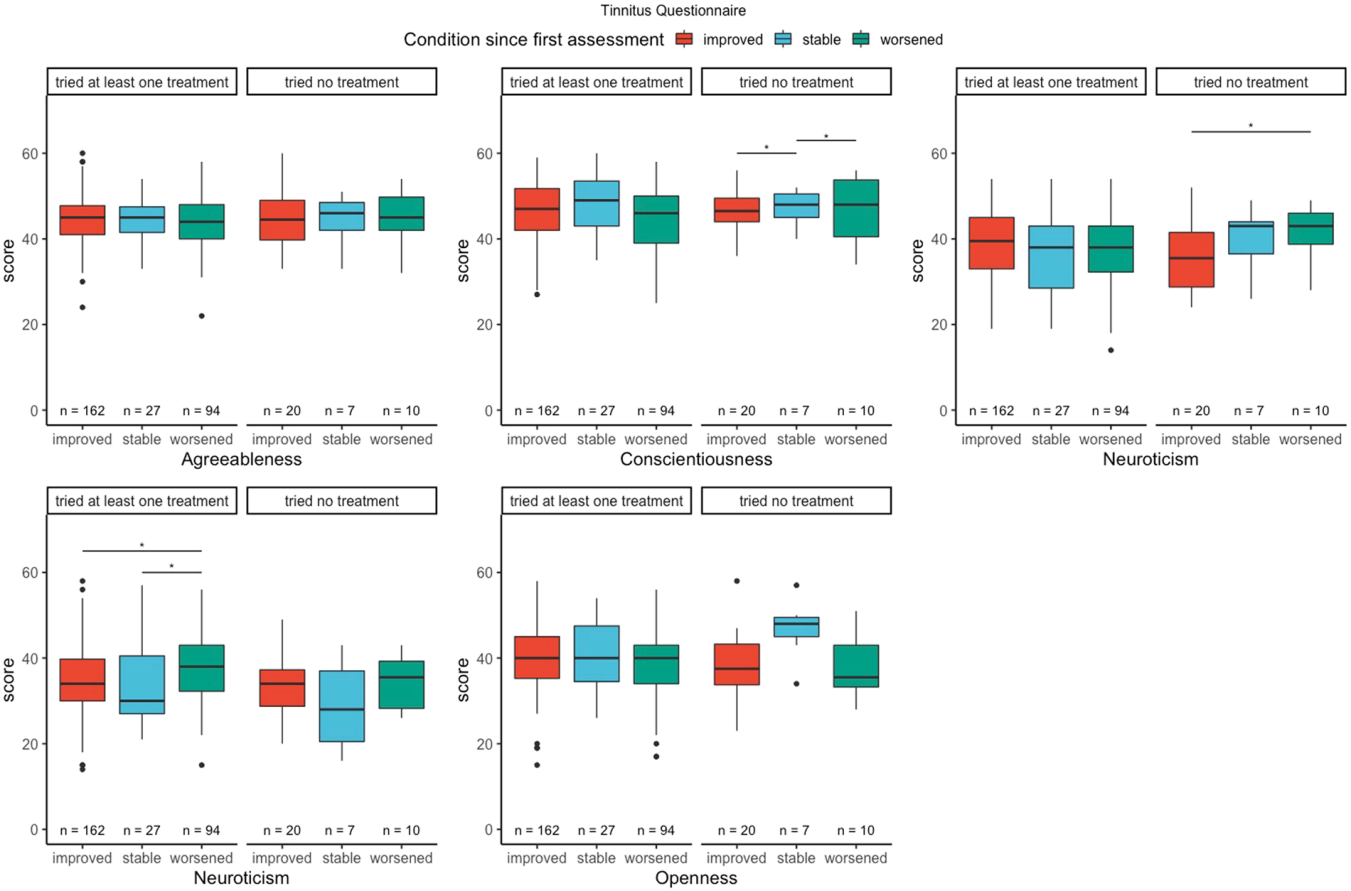
TQ change grades and personality traits. Results are reported for patients who tried at least one treatment and patients who tried no treatment.

For this analysis, we clustered patients in two groups: patients who tried at least one tinnitus-related treatment between T1 and T2, and patients who did not. Results are presented in Figs. 4 and 5. Patients who did try at least one treatment between T1 and T2, and binned in the “improved” group for the THI scored lower in neuroticism than patients binned in the “worsened” group (t = −2.96, p-value < 0.01, d = −0.47). Similar results were observed with the TQ (t = −2.13, p = 0.03, d = −0.27). Regarding extraversion, we observed statistical significant differences between the “improved” and “stable” groups (t = 2.68, p-value < 0.01, d = 0.35), and between the “improved” and “worsened” group (t = 2.05, p-value = 0.04, d = 0.34) for the THI in the group of patients who did not try any treatment between T1 and T2. For the TQ, we observed a statistical significant change between the groups “improved” and “worsened” among patients that tried at least one treatment between T1 and T2 (W = 51, p-value = 0.03).

## Discussion

The present study was the first to systematically analyze the effect of personality on the longitudinal trajectory of tinnitus distress. We showed that neuroticism is related to changes in tinnitus distress, i.e., clinically relevant changes in the grade of tinnitus distress as measured by TQ and THI. More specifically and of central interest, neuroticism was higher in patients with worsened clinical status compared to patients with improved clinical status (measured with THI as well as TQ). Looking at extraversion, the group showing clinical improvement exhibited significantly higher scores compared to the groups of stable and worsened clinical status (measured with THI but not significant for TQ).

Our cross-sectional analysis between personality and tinnitus distress reproduced previous findings^13,14,19,20^, as both neuroticism and agreeableness showed a positive association with tinnitus distress, while extraversion had a negative association with distress. We partially reproduced the findings of Strumila and colleagues, who identified neuroticism as the sole predictor of the THI^17^, which could be explained by differences in the demographics of both samples 2.

We identified the three facets of neuroticism, anxiety, depression and emotional volatility, to be significant predictors of the THI and TQ cross-sectionally. We also identified energy level, a facet of extraversion, to be a a significant predictor of the THI. Interestingly, those facets are characteristic of the “type D” personality, which is prevalent among high distressed tinnitus patients^14,16^. A previous review^19^ discussed the challenges of separating depression and tinnitus, as the symptoms presented by both groups and the underlying mechanisms between the two conditions are often convoluted (e.g., neuronal mechanisms^21^). Future studies could investigate the relation between the two conditions by comparing acute depression (e.g., as measured by the Beck’s or Major Depression Inventory), depressive personality (e.g., as measured by the “depression” facet) and biomakers of depression, such as the brain-derived neurotrophic factor^22^.

We could not observe a significant effect of any of the five personality traits on tinnitus distress over time in our multiple linear regression setup (except conscientiousness, which was at the margin of statistical significance in both models). On the other hand, the year of first assessment was a significant predictor of the difference in the THI and TQ in T2 − T1, suggesting that the longer the period between T1 and T2, the greater the decrease in tinnitus distress. To test for clinical relevance, which surpasses mere psychometric statistical analysis and therefore generate important practical insights for clinical routine in tinnitus, we grouped patients in three groups based on previous literature^23,24^: “clinically improved”, “clinically stable”, and “clinically worsened”.

These differences reflected the difference in the THI and TQ between two time points. Patients grouped in the “worsened” group had higher levels of neuroticism than the other two groups in both the THI and the TQ, and extraversion was significantly higher in the “improved” group than in the other two for the differences in the THI, but not in the TQ. These differences in personality related to clinically relevant changes in tinnitus distress may have prognostic value, as they were able to statistically distinguish three clinically relevant groups of tinnitus patients. Similar effects were observed when we divided the three groups between patients who tried at least one clinical trial for their tinnitus and those who did not. Our results suggests that personality plays a role on the change of distress among patients who tried at least one treatment. There is an on going debate about the role of personality on placebo effects in clinical trials, and researchers are increasingly aware of its putative effects alongside other factors such as positive/negative expectations and patient-clinician relationship^25^. We recommend future tinnitus studies to measure and report personality traits in their clinical trials, as personality could either represent a cofounder in trials’ outcomes or be used to identify efficient individual treatments.

Tinnitus heterogeneity has been implicated as a major obstacle to improving the condition’s management, and thus tinnitus subtyping has been settled as a major objective in the research community^4,5^. Future research should further explore the role of personality on tinnitus heterogeneity and its implications on treatment outcomes. For instance, although the relation between personality and coping strategies is well characterized in the overall literature^26^, little is known about it in tinnitus patients.

This study has inherent limitations. First, we collected the FFM at T2 and not at T1. Whether personality is a crystallized construct, ie., it does not change over time, is debatable. For instance, it is possible to change FFM scores through experimental manipulation^27^. Conversely, Cost and Mccrae^11,12^ reviewed the evidence for the stability of the construct longer periods of time. The stability of personality over time, in our cohort up to seven years to patients who visited our clinic in 2012, is an important assumption of our analysis^12^. As proposed above, it would therefore be helpful to assess personality within standard psychometric tinnitus batteries at all time points in clinical trials or general longitudinal research. Second, we could not properly investigate the role of personality among patients who did not try any treatment due to small sample size. Whether clinically significant tinnitus habituation can be explained by personality remains a relevant, open question. Third, we could not discard a potential bias among those who responded to our survey at T2. To the best of our knowledge, no former study investigated whether patients participate in surveys and/or clinical trials. However, apart from a 2-point difference in the TQ, we could not find statistically significant differences between the sample which responded the survey and the sample which did not.

In conclusion, our results suggest that personality traits, namely neuroticism and extraversion, can explain a large portion of the variance of tinnitus distress. Those two traits are relevant markers of tinnitus distress over time and can be used to statistically distinguish patient groups with clinically relevant changes of tinnitus distress. Personality assessments could provide valuable information to clinicians and researchers, and may eventually be used to deliver personalized treatments for tinnitus patients^28^. Future studies would furthermore profit from assessing personality at several time points to further investigate interactions between personality and tinnitus.

## Methods

### Participants

Previous patients of the Tinnitus Outpatient Center of the University of Regensburg were invited to participate in this questionnaire survey by a letter with questionnaires and consent forms. 1213 letters were sent to patients who visited the clinic between 2012 and 2017, from which 388 sent back with signed consent forms. The study was approved by the ethical committee at the faculty of medicine of the University of Regensburg (Study Number 18-1041-101), and all methods were performed in accordance with the relevant guidelines and regulations of the institution. We obtained written informed consent from all participants who agreed on having their anonymized data stored and used for scientific purposes.

### Data collection

The longitudinal analysis considered the first questionnaire assessment during the visit to the clinic as T1 and the assessment with the questionnaires delivered by mail in 2018 as T2. The THI, TQ and TSCHQ were collected at T1 and T2, and the BFI-2 and an informal in-house questionnaire asking patients which treatments they tried between T1 and T2. Regarding the BFI-2, patients filled in a previously validated German version of the questionnaire^29^.

### Statistical analysis

To evaluate the potential predictive role of the BFI2 on tinnitus distress over time, we grouped patients in three categories: clinically “improved”, “stable”, and “worsened”. The groups reflect the difference of the THI and TQ scores as the difference in scores between the two time points, and followed the guidelines of Zeman *et al*., and Adamchic *et al*.^23,24^. More specifically, for the THI, a decrease in >7 points at T2 was considered a significant clinical improvement, an increase in >7 was considered a clinical significant worsening, and the patients who did not score at T2 higher or lower than seven points were grouped as “stable”. For the TQ, the thresholds for improvement and worsening were, respectively, 5 points lower and 1 point higher in T2 compared to T1. Since T1 spanned between 2012 and 2017, the year of visit T1 was treated as covariate in the longitudinal analysis. All statistical analyses were conducted with R statistical software (version 3.4.4, R Development Core Team, 2008), alongside the “tidyverse” package^30^. Effect sizes were calculated with the package “effsize^31^”, and the panels with figures were generated with the “ggpubr” package. Non-parametric tests were used when test assumptions were not met. P values below 0.05 were considered statistically significant.
